# *Fasciola hepatica* induces Foxp3 T cell, proinflammatory and regulatory cytokine overexpression in liver from infected sheep during early stages of infection

**DOI:** 10.1186/s13567-018-0550-x

**Published:** 2018-07-03

**Authors:** Isabel L. Pacheco, Nieves Abril, Rafael Zafra, Verónica Molina-Hernández, Noelia Morales-Prieto, María J. Bautista, María T. Ruiz-Campillo, Raúl Pérez-Caballero, Alvaro Martínez-Moreno, José Pérez

**Affiliations:** 1 Department of Anatomy and Comparative Pathology, Faculty of Veterinary Medicine, University of Córdoba, Sanidad Animal Building, Rabanales Campus, Córdoba, Spain; 2 Department of Biochemistry and Molecular Biology, Faculty of Sciences, University of Córdoba, Severo Ochoa Building, Rabanales Campus, Córdoba, Spain; 3 Department of Animal Health (Parasitology), Faculty of Veterinary Medicine, University of Córdoba, Sanidad Animal Building, Rabanales Campus, Córdoba, Spain

## Abstract

The expression of T regulatory cells (Foxp3), regulatory (interleukin [IL]-10 and transforming growth factor beta [TGF-β]) and proinflammatory (tumor necrosis factor alpha [TNF-α] and interleukin [IL]-1β) cytokines was quantified using real time polymerase chain reaction (qRT-PCR) in the liver of sheep during early stages of infection with *Fasciola hepatica* (1, 3, 9, and 18 days post-infection [dpi]). Portal fibrosis was also evaluated by Masson’s trichrome stain as well as the number of Foxp3^+^ cells by immunohistochemistry. Animals were divided into three groups: (a) group 1 was immunized with recombinant cathepsin L1 from *F. hepatica* (FhCL1) in Montanide adjuvant and infected; (b) group 2 was uniquely infected with *F. hepatica*; and (c) group 3 was the control group, unimmunized and uninfected. An overexpression of regulatory cytokines of groups 1 and 2 was found in all time points tested in comparison with group 3, particularly at 18 dpi. A significant increase of the number of Foxp3^+^ lymphocytes in groups 1 and 2 was found at 9 and 18 dpi relative to group 3. A progressive increase in portal fibrosis was found in groups 1 and 2 in comparison with group 3. In this regard, group 1 showed smaller areas of fibrosis than group 2. There was a significant positive correlation between Foxp3 and IL-10 expression (by immunohistochemistry and qRT-PCR) just as between portal fibrosis and TGF-β gene expression. The expression of proinflammatory cytokines increased gradually during the experience. These findings suggest the induction of a regulatory phenotype by the parasite that would allow its survival at early stages of the disease when it is more vulnerable.

## Introduction

Fasciolosis, caused by *Fasciola hepatica*, produces high economic losses to the agricultural sector [1]. This parasite infects a wide range of domestic animals including cattle, sheep, and goats, and the disease has been recognized as an important zoonosis in Africa, Asia, Europa, America, and Oceania [2, 3]. Traditionally, the control of the disease has been based on the use of anthelmintic drugs. Nevertheless, resistance to triclabendazole and other drugs as well as the public concern about the presence of drug metabolites in foodstuff is increasing in numerous countries [4]. Due to this, during the last few years an enormous interest in the development of an immunological method to control the disease has risen [5, 6]. Despite major efforts during the last two decades, the search for an effective vaccine to control this disease has been slow. This is due to the different mechanisms used by *F. hepatica* to modulate the host immune response making it ineffective to kill the parasites [7–9]. One of these mechanisms, like for other helminths, is the expansion of T regulatory cells (Foxp3), facilitating both parasite survival and modulation of tissue damage [10, 11]. Our group has recently shown the expansion of Foxp3^+^ T cells in the liver and hepatic lymph nodes of sheep and goats infected with *F. hepatica* [12].

In previous studies, it has been reported that *F. hepatica* is able to downregulate the Th1 immune response and upregulate the Th2 response at early stages of infection in sheep [13] and mice [14] as well as in chronic stages in cattle [15]. This imbalance towards a Th2 immune profile is mediated through regulatory cytokines and cells that modulate and/or suppress inflammatory responses. The induction of a regulatory environment by the expression of cytokines such as IL-10 and TGF-β has been shown as a common strategy used by parasites and microorganisms to extend their survival [16–18]. As a consequence of this regulatory environment, the expression of Foxp3 T cells is increased. Specifically, in *F. hepatica* infection it has been shown that Foxp3^+^ lymphocytes play an important role contributing to the parasite survival during the migratory stage [10, 12]. In addition, *F. hepatica* develops other mechanisms to evade the host’s immune response in early stages in sheep where larvae are able to induce apoptosis of peritoneal leukocytes, allowing the migration of larvae through the peritoneum [19].

In rats, the protective response against *F. hepatica* has been reported during initial stages of infection [20]. On the other hand, an increase in inducible nitric oxide synthase (iNOS) expression in peritoneal leukocytes has also been reported in goats, suggesting that eosinophils may play an important role in the host response during early stages of infection [21]. For all these reasons, it is of crucial importance to study the host’s immune mechanisms at this stage of infection when the parasite seems to be more vulnerable to the immune response.

The aim of this study was to evaluate the gene expression of regulatory cytokines (IL-10 and TGF-β), proinflammatory cytokines (TNF-α and IL-1β), the transcription factor Foxp3 at different levels (gene and antigenic expression), and portal fibrosis in liver tissue samples from unimmunized and immunized (recombinant cathepsin L1 -FhCL1) sheep during early stages of the infection with *F. hepatica*.

## Materials and methods

### Experimental design

The experimental design has been described previously by our group [13]. Briefly, 44 seven-month old female Merino-breed sheep obtained from a liver fluke-free farm were used for this study. All animals were tested monthly for parasite eggs by fecal sedimentation with negative results in all cases. Moreover, prior to the challenge, all animals were tested for serum IgG specific for *F. hepatica* cathepsin L1 (FhCL1) by enzyme-linked immunosorbent assay ELISA, with negative results in all cases. Animals were housed indoors (100 m^2^ covered and 100 m^2^ uncovered facility) and fed with hay and pellets and water ad libitum. The sheep were distributed into three groups: group 1 (*n* = 20) was immunized subcutaneously with two doses 4 weeks apart with 100 µg rCL1 in 1 mg of Montanide ISA 70 VG (Seppic, Puteaux, France). Recombinant FhCL1 was kindly provided by Prof. John Dalton (Queen’s University Belfast, Belfast, UK). group 2 (*n* = 20) was unimmunized and infected. group 3 (*n* = 4) was unimmunized and uninfected.

Groups 1 (immunized and infected) and 2 (unimmunized and infected) were orally infected with 200 metarcercariae of the South Gloucester strain of *F. hepatica* (Ridgeway Research Ltd, UK) and divided into four subgroups each (*n* = 5), which were euthanized five animals from group 1 and five animals from group 2 at 1, 3, 9, and 18 days post-infection (dpi). Ten sheep were sacrificed daily (5 from group 1 and 5 from group 2). In all animals, the euthanasia was conducted by intravenous injection of 7 mL of Embutramide 200 mg and Mebezonium iodide 50 mg. No adverse reactions or clinical signs were noted during the experiments. The experiment was approved by the Bioethics Committee of the University of Cordoba (No. 1118) and conducted in accordance with European (2010/63/UE) and Spanish (RD 1201/2005) directives on animal experimentation. The vaccine was non-protective as reported in a previous work [13].

### Histopathology

At necropsy, the liver was removed and photographed on the visceral and diaphragmatic aspects for gross evaluation. Liver tissue samples were collected and samples frozen for qRT-PCR examination, while other samples were fixed in 10% neutral buffered formalin for 24 h, then routinely processed and embedded in paraffin wax. Four micron-thick tissue sections were stained with hematoxylin and eosin (H&E) for histopathology. Masson’s trichrome staining (Bio-Optica, Milan, Italy) was used to assess portal fibrosis in liver tissue slides.

### Immunohistochemical analysis

An immunohistochemical study was used to assess Foxp3 expression in liver tissue samples. The avidin–biotin–peroxidase method described elsewhere [12] was carried out. Briefly, after hydration of samples, antigen retrieval was carried out by incubating the slides in citric acid (pH 6.0) followed by heating in an autoclave for 10 min at 121 °C. Samples were rinsed twice for 5 min in phosphate buffered saline (PBS) and 5 min in PBS-Tween 80 (Panreac, Barcelona, Spain). Endogenous peroxidase activity was blocked by incubation with 0.3% hydrogen peroxide (Panreac, Barcelona, Spain) in PBS-Tween 80. The slides were then rinsed twice for 5 min in PBS-Tween 80 and incubated with 25% normal goat serum (Vector Laboratories, Burlingame, California, USA) for 1 h at room temperature. An anti-mouse/rat Foxp3 monoclonal antibody (clone FJK-16s, rat IgG2a, eBioscience Inc. San Diego, CA, USA) was diluted 1:100 in PBS containing 10% normal goat serum and applied to the slides overnight as primary antibody. Then, two rinses in 10% normal goat serum were performed prior to a goat anti-rat immunoglobulin serum (Vector Laboratories, Burlingame, California, USA) diluted 1:100 that was applied for 30 min. After three rinses of 7 min in PBS an avidin–biotin–peroxidase complex (Vector Laboratories, Burlingame, California, USA) diluted 1:50 was applied for 1 h. Tissue sections were then washed three times in Tris-buffered-saline (TBS, pH 7.2) and incubated with the vector NovaRED^®^ peroxidase substrate kit (Vector Laboratories, Burlingame, California, USA) for 5 min. Then, samples were rinsed in tap water, lightly counterstained with Mayer’s hematoxylin and mounted with Eukitt^®^ (Freiburg, Germany). Specific primary antibodies were substituted with PBS or non-immune isotype-matched sera as negative control. Hepatic lymph node sections from sheep were used as positive controls.

### Morphometry and cell counting

Both portal fibrosis evaluation and Foxp3^+^ cell counting was done using the biomedical software Image J v.1.51d. In order to measure the fibrotic areas, three liver slides per animal were used and five random microphotographs at 200× magnification were taken for each slide. It was necessary to develop specific macros to calibrate the measure and intensity of positive areas (expressed in µm^2^) for Masson’s trichrome staining. The number of Foxp3^+^cells were carried out using three liver slides per animal and five random microphotographs at 400× magnification. In this case, the specific macros developed were carried out to calibrate the appropriate immunostaining intensity and cell size. In both cases, the results were expressed as mean ± SD.

### RNA extraction and cDNA synthesis

Samples collected from the left liver lobe were washed in diethylpyrocarbonate (DEPC) biomolecular water and immediately frozen in liquid nitrogen, individually disrupted in liquid nitrogen, and finally stored at −80 °C. Total RNA was isolated from 300 mg of ground tissue homogenized in 1.5 mL of TRIzol^®^ reagent (Ambion Life Technologies, Carlsbad, CA, USA) using a sterilized IKA^®^T10 basic disperser and then cleaned with the RNeasy^®^ Mini Kit (Qiagen, Hilden, Germany) according to manufacturer’s guidelines. Incubation with RNase-free DNase I (Qiagen, Hilden, Germany) for 15 min was included in the protocol. Isolated total RNA was finally incubated at 65 °C for 10 min and kept at −80 °C until used. Concentration and purity of RNA were determined by spectrophotometry. The Agilent 2100 Bioanalyzer (Agilent Technologies, Santa Clara, CA, USA) was used to determine the RNA integrity number (RIN), whose values range from 0 for degraded RNA to 10 for intact RNA [22]. The quality criteria to use RNA samples in the qRT-PCR experiment were: (1) RIN values ≥ 8.5; (2) ratios 260/280 about 2; and (3) absence of gDNA. The iScript™ cDNA Synthesis Kit (BioRad, Hércules, CA, USA) was used to generate cDNAs from 1 μg of total RNA from each sample individually.

### Primer design

The primer pairs for gene expression analysis of the targets Foxp3, IL-10, TGF-β, TNF-α, and IL-1β in liver tissue were designed with Oligo 7 software (Colorado Springs, USA) over specific sequences obtained from the GenBank database. In all cases, primer sizes were between 25 and 30 base pair (bp) and lengths of generated amplicons ranged from 99 to 192 bp. To secure primer specificity for target cDNA, the sense and antisense primers were located in different close exons. To obtain high specificity and better performance, primers devoid of hairpin and duplex structure were required to have a high temperature (≥ 68–70 °C) and optimal 3’−∆G (≤ −6 kcal/mol) values for use in two-step 94/68 °C PCR. All primer pairs produced amplicons of the predicted size (Table 1). All PCR products were further verified by nucleotide sequencing.

**Table 1:** Description and sequences of the oligonucleotides designed to quantify specific ovine genes using real-time PCR

Genes	Sequences	Amplified product size (bp)	Accession number
Foxp3	F 5′-GCCCATCTGGCTGGGAAGATGGCCCAAACC-3′	166	NM_001144947.1
	R 5′-AGAGGTGCCTCCGCACGGCAAACAGG-3′		
IL10	F 5′-TCAGCCGTGCTCTGTTGCCTGGTCTTCC-3′	124	NM_001009327.1
	R 5′-GGACGTCCCGCAGCATGTGGGGCAG-3′		
TGFβ	F 5′-GGGCTTTCGCCTCAGTGCCCACTGTTC-3′	151	NM_001009400.1
	R 5′-CAGAGGGGTGGCCATGAGGAGCAGG-3′		
TNFα	F 5′-CCACGCTCTTCTGCCTGCTGCACTTCGG-3′	146	NM_001024860.1
	R 5′-AACGTGGGCTACCGGCTTGTTATTTGAGGC-3′		
IL1β	F 5′-GAAGCTGAGGAGCCGTGCCTACGAACA-3′	185	NM_001009465.2
	R 5′-CCAGCACCAGGGATTTTTGCTCTCTGTCC-3′		

### Absolute quantitation of cytokine transcripts by real-time PCR

**Figure 1:**
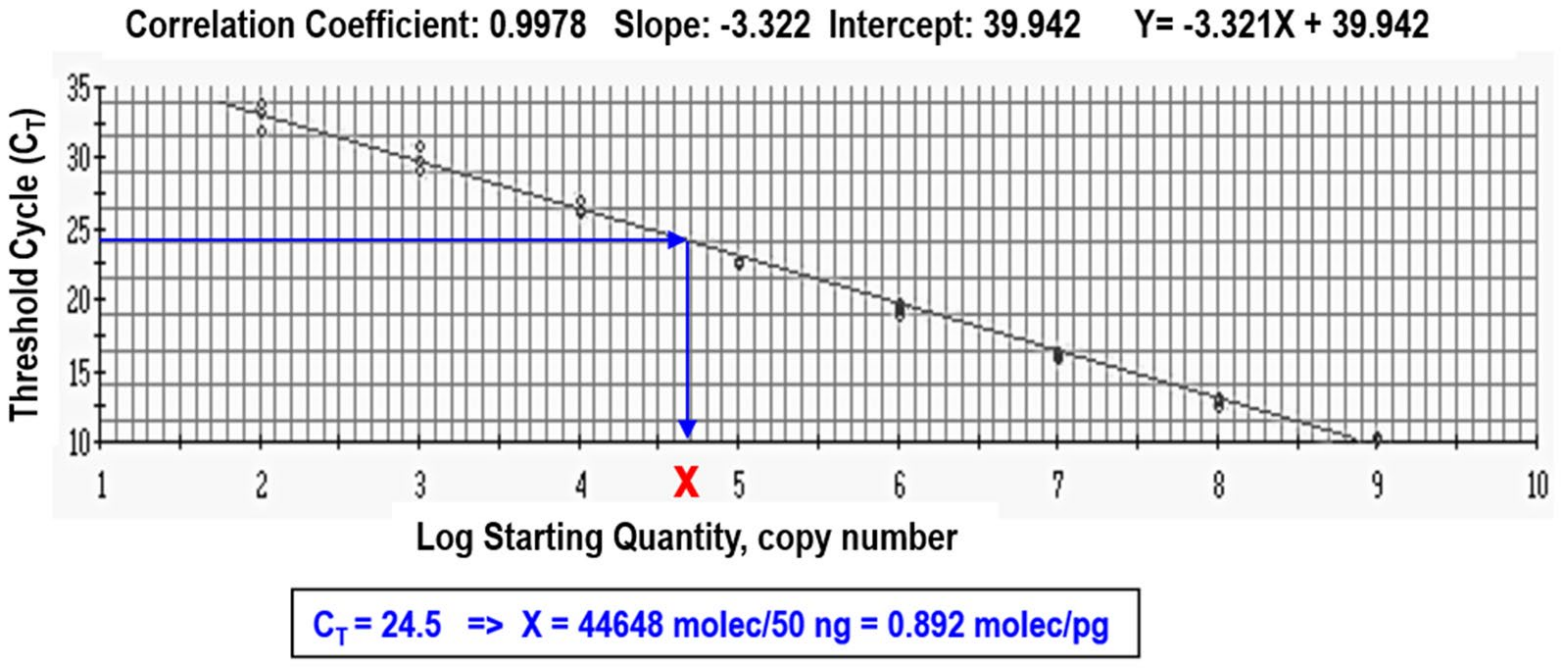
**Absolute standard curve used to calculate the number of copies per pg of total RNA of each mRNA.** The absolute standard curve was prepared with an in vitro synthesized RNA corresponding to a 457-nt fragment of the mouse *Gapdh* transcript (GenBank Database: M32599). Concentration was spectrophotometrically determined by A260 and converted to the number of copies using the molecular weight of the RNA fragment. Serial dilutions (10^9^ to 10^2^ RNA copies) were prepared, retrotranscribed, and amplified by real-time PCR. Primers have been previously described [23]. The standard curve was constructed by plotting the log of starting RNA molecules versus the threshold cycle (Ct). The resulting standard curve is linear (r = 0.998) over 7 orders of magnitude. The efficiency (E) value is calculated from the slope of the standard curve equation, as E = 10^[− 1/slope]^− 1. The slope of the standard curve indicates that the standard is amplified with 100% efficiency. This standard curve was used to determine the number of copies of each experimental transcript, as exemplified for a Ct = 24.5.

The SsoAdvanced™ Universal SYBR^®^ Green Supmermix (BioRad) Kit was used in real-time PCR according to manufacturer’s guidelines. Amplifications were performed in triplicate by using 50 ng of cDNA from each animal and 0.3 µM of each primer in a MyiQ™2 Two Color Real-Time PCR Detection System (BioRad). Cycling conditions consisted of 2 min at 95 °C for Platinum Taq activation followed by 40 two-step cycles for melting (15 s, 95 °C), and annealing/extension (30 s, 70 °C). After 40 cycles, a melting curve analysis was performed (60‒95 °C) to verify the specificity of amplicons. Replicate PCR generated highly reproducible results with standard error of the mean (SEM) < 10% of the mean (< 1% for threshold cycle data). All targets amplified with the same optimal PCR efficiency (100%) and high linearity (r > 0.99) in the range of 20 to 2 × 10^5^ pg of total RNA input. An inter-run calibrator (IRC) RNA sample, with a known number of transcripts of the A170 gene, was introduced in each experiment to guarantee the quality of the retro-transcription and to detect and remove inter-run variation. An absolute calibration curve was generated with an in vitro transcribed RNA containing a known number of copies (Figure 1), as previously described [23, 24]. The number of transcript molecules corresponding to each experimental gene was calculated from the linear regression of the calibration curve.

### Statistical analysis

The number of mRNA molecules per µg RNA total are shown with averages and SEM. Comparisons of variables between control and infected groups were carried out by using Student’s t test followed by Bonferroni correction for multiple comparisons. For both immunohistochemical and morphometrical studies the results were expressed as mean ± standard deviation (SD). The Kolmogorov–Smirnov test was applied to evaluate if data were normally distributed. Data were analyzed with the non-parametric Kruskall–Wallis multiple comparison test with Dunn’s post hoc test. Correlation studies were estimated using the Spearman’s non-parametric correlation test. For all the statistical tests, significance was stablished with a *P* value < 0.05. The statistics software used were Sigma Stat 5.1 (San Jose, CA, USA) and GraphPad Prism 7.0 (GraphPad Software, Inc., San Diego, CA, USA).

## Results

### Liver pathology

Gross hepatic lesions of groups 4 and 6 were typical lesions of chronic fasciolosis: tortuous whitish tracts and hemorrhagic spots, mainly affecting the left hepatic lobe. The severity of gross hepatic lesions from groups 4 and 6 was similar. Gross and histopathological hepatic changes in groups 1 and 2 have been reported previously, with the only significant difference between both groups at 9 dpi when group 1 (immunized and infected) showed lower hepatic lesions than group 2 (unimmunized and infected) [13].

**Figure 2:**
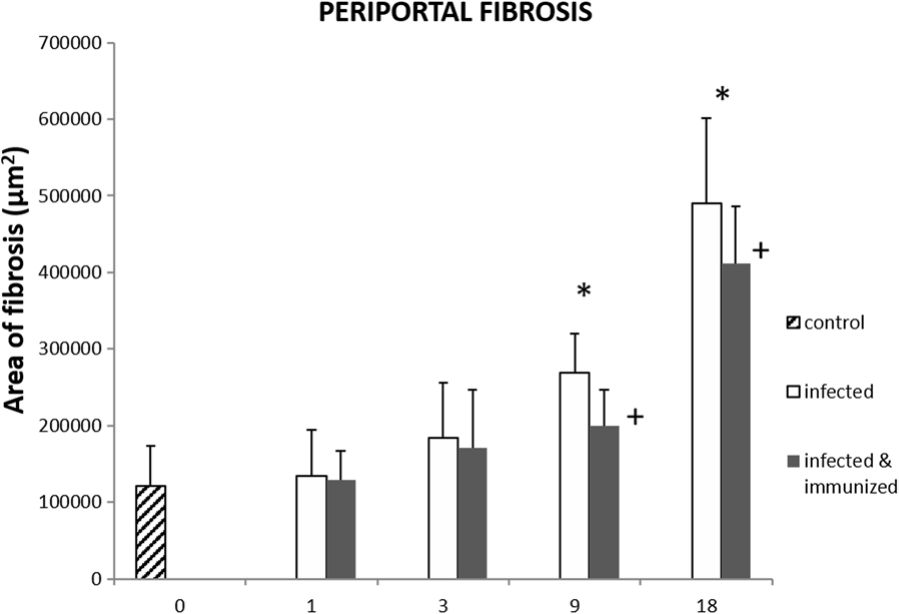
**Comparison of areas with periportal fibrosis stained with Masson’s trichrome.** There was a gradual increase of periportal fibrosis across the different dpis, being statistically significant at 9 and 18 dpi (*) with respect the negative control group (*P* < 0.0001). The immunized animals showed slightly less fibrosis than those infected at 1 and 3 dpi. However, this decrease was significant at 9 and 18 dpi (+) (*P* < 0.0001).

**Figure 3:**
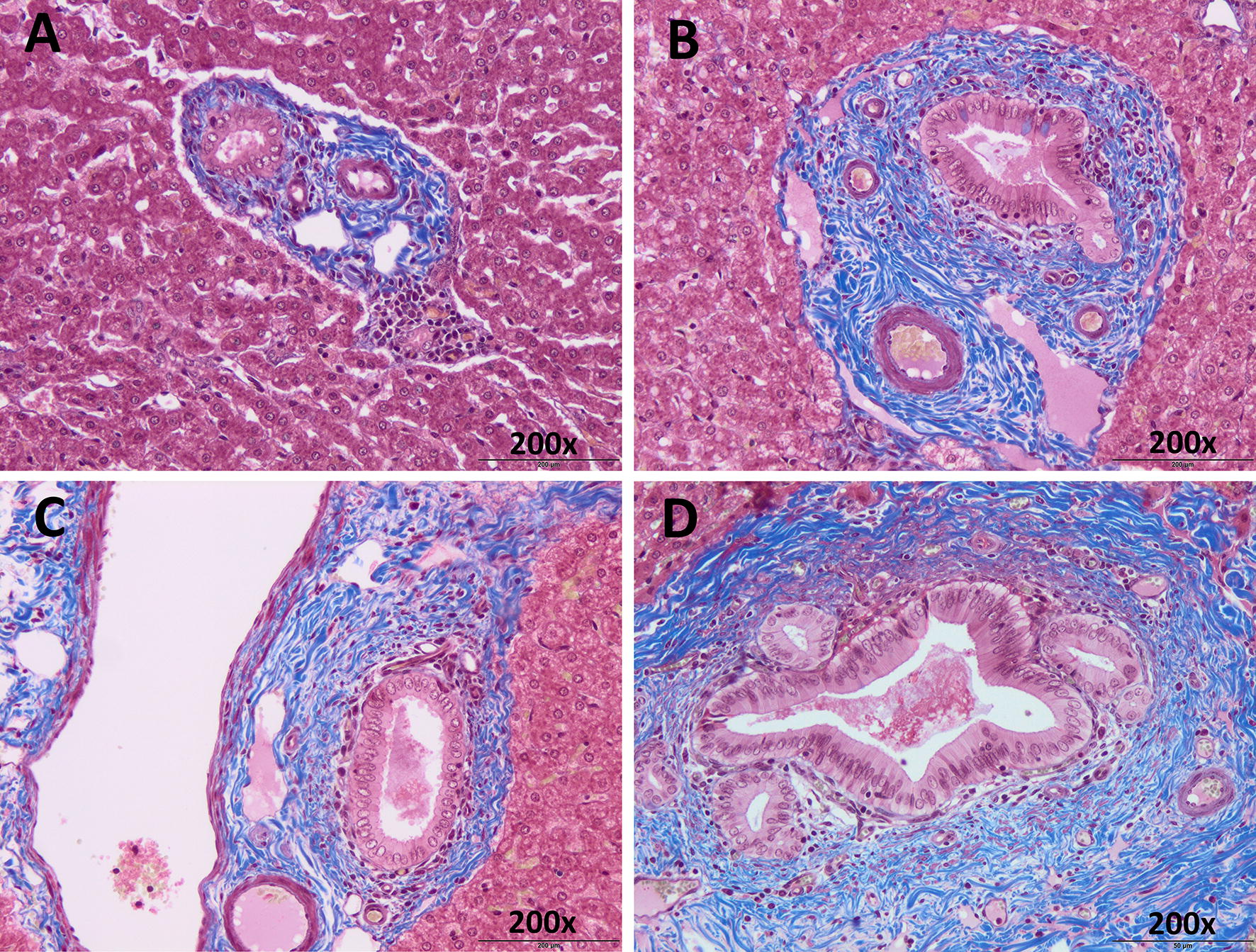
**Liver, microphotographs of periportal fibrosis. A** Representative picture of periportal fibrosis at 1 and 3 dpi either infected or immunized group. **B** Periportal fibrosis produced in animals from the infected group at 9 dpi. **C** Periportal fibrosis of the animals belonging to the immunized group at 18 dpi. **D** Periportal fibrosis of animals from the infected group at 18 dpi. Masson’s trichrome staining, ×200.

Results of fibrosis measurements in groups 1, 2 and 3 are summarized in Figure 2. A gradual increase of hepatic fibrosis through the experiment was found. This increase was statistically significant (*P* < 0.0001) in both groups infected (Figure 3) at 9 and 18 dpi compared with group 3 (unimmunized and uninfected). At this timepoint, group 2 (unimmunized and infected) showed higher areas of portal fibrosis than group 1 (immunized and infected) (Figure 2).

### Immunohistochemistry

**Figure 4:**
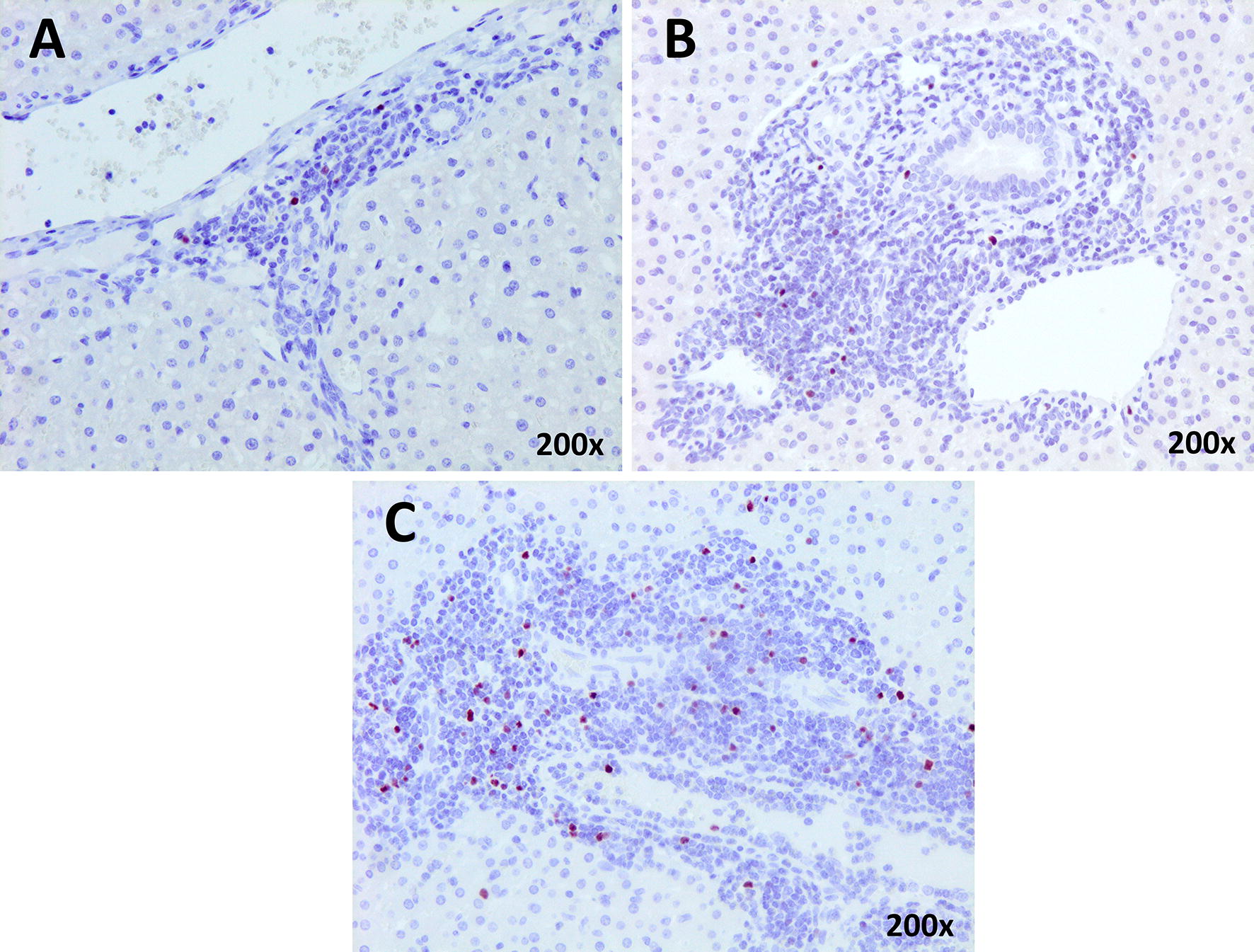
**Liver, microphotographs of positive Foxp3 cells. A** Positive Foxp3 cells found in both groups, immunized and infected at 1 and 3 dpi. **B** Positive Foxp3 cells found in the immunized group at 18 dpi. **C** Positive Foxp3 cells found at 9 dpi and the infected group at  dpi. ABC-hematoxylin counterstain, ×200.

**Figure 5:**
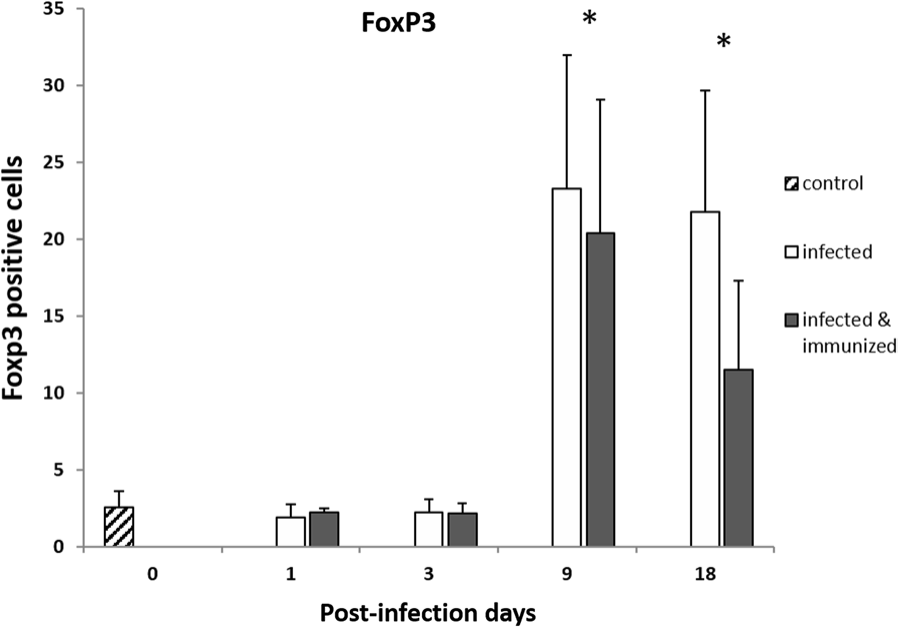
**Comparison between different groups of positive Foxp3 cells in immunohistochemistry.** A significant increase in the expression of Foxp3+ lymphocytes was observed in the infected control group at 9 and 18 dpi (*) with respect to the negative control group and respect to the infected control and immunized groups at 1 and 3 dpi (*P* < 0.0001). No significant differences were found between the infected control group and the immunized group during the experiment.

The anti-Foxp3 mAb yielded a nuclear and/or cytoplasmic immunostaining in lymphocytes mainly located in portal areas (Figure 4) and in a lesser amount in the periphery of granulomas and necrotic foci. The results of the immunohistochemical study of Foxp3 in liver are summarized in Figure 5. There were no significant differences between groups in Foxp3^+^ cell expression at 1 and 3 dpi. However, a significant increase (*P* < 0.0001) of Foxp3^+^ cells was observed at 9 and 18 dpi in groups 1 and 2 compared with group 3. The statistical analysis revealed a strong positive correlation between the number of Foxp3^+^ T cells in the hepatic inflammatory infiltrates and IL-10 gene expression (r = 0.81; *P* = 0.0004 and r = 0.88; *P * < 0.0001 for groups 1 and 2, respectively).

### Cytokine gene expression

**Figure 6:**
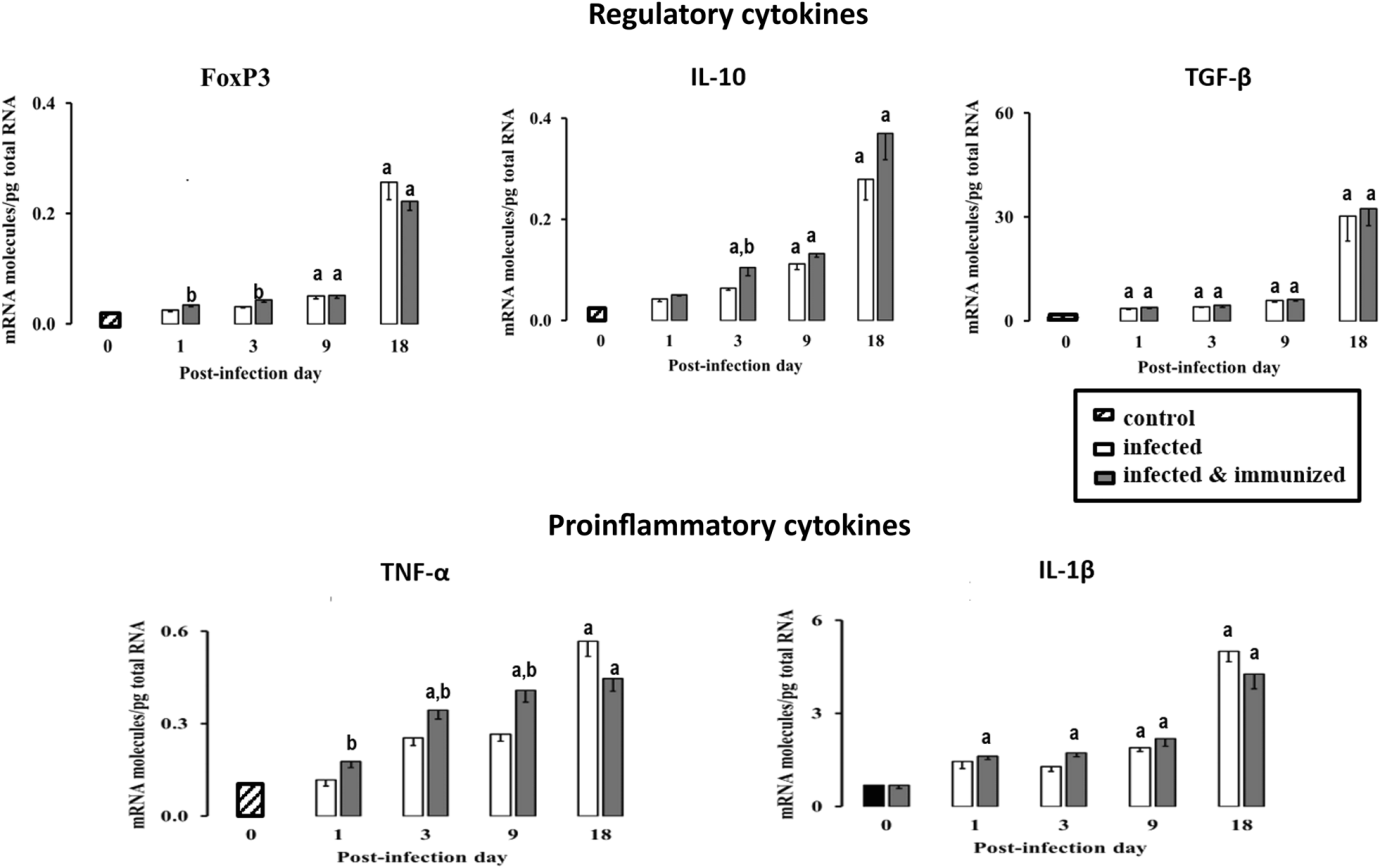
**Gene expression of Foxp3, IL-10, TGF-β, IL-1β, and TNF-α.** Each bar represents the mean ± SD of the mRNA molecules/ng of total RNA quantified individually in each of the five animals per experimental condition and sampling time, after three real-time PCR reactions per individual. *a* for comparison of each treatment with the negative control, and *b* for comparisons between the infected and infected and immunized groups. The levels of gene expression were increasing gradually throughout the experiment in the transcription factor, foxp3 and two regulatory cytokines studied (IL-10, TGF-β), reaching its higher expression at 18 dpi, and for IL-10 also markedly at 3 and 9 dpi, coinciding with a slight increase in the expression of Foxp3. Foxp3, IL-10, and TGF-β showed significant differences in comparison with the negative control group at 1, 3, and 9 dpi, respectively. At the same time, differences were shown between immunized and infected groups at 1 and 3 dpi in Foxp3 and 3 dpi in IL-10. The expression of proinflammatory cytokines (IL-1β and TNF-α) was gradually increasing along the experience, showing the typical expression of an acute stage of disease.

Gene expression levels in liver for Foxp3, IL-10, TGF-β, TNF-α and IL-1β are shown in Figure 6. During the course of infection, Foxp3 expression did not change significantly at 1 and 3 dpi in any of the groups analyzed. Nevertheless, at 18 dpi, groups 1 and 2 showed a significant increase (*P* < 0.001) in Foxp3 expression compared to group 3. Foxp3 gene expression was higher at 1, 3, and 9 dpi in group 1 compared with group 2. At 18 dpi, group 2 showed higher expression of Foxp3 with respect to the counterparts immunized (Figure 6). There were no statistically significant differences between groups 1 and 2. IL-10 RNA expression increased gradually in both infected groups with respect to the uninfected. This increase was significant from 9 dpi onwards (*P* < 0.001) in the infected control group and from 3 dpi onwards (*P* < 0.001) in the infected and immunized group (Figure 6). The immunized and infected group showed higher IL-10 expression than the infected group through all timepoints. Nevertheless, this IL-10 expression increase only was statistically significant at 3 dpi (*P* = 0.03). There was a significant positive correlation between IL-10 and Foxp3 gene expression for the immunized group (r = 0.68; *P* = 0.0002) and infected group (*P* < 0.0001; r = 0.81), respectively.

Gene expression levels for TGF-β showed a significant increase (*P* < 0.001) in both infected groups at 1, 3, 9, and 18 dpi compared to the uninfected (Figure 6). This increase reached the maximum level at 18 dpi in both infected groups, and the increase was also significantly higher (*P* < 0.001) than in previous infection stages. There were no differences between the infected group and the immunized and infected group through the experience. The correlation found between TGF-β expression and portal fibrosis also showed a positive correlation for both immunized (*P* = 0.017; r = 1) and infected (*P* = 0.017; r = 1) groups.

TNF-α expression exhibited a gradual increase throughout all timepoints. Both infected groups showed a significant increase (*P* < 0.05) in TNF-α expression in comparison with the uninfected and unimmunized group (group 3), except for the infected/unimmunized group at 1 dpi. Between infected groups, the immunized (group 1) showed a significant increase in the expression of TNF-α at 1, 3, and 9 dpi (*P* < 0.05) compared with the infected (Figure 6). However, at 18 dpi, the expression was inverse, showing a significant increase (*P* < 0.001) in the infected group at this timepoint in comparison with the immunized and infected group.

Finally, the expression of IL-1β gradually increased through the experience for both infected groups. This increase was significant at all timepoints compared to the uninfected and immunized group (*P* < 0.01) and from 9 dpi onward in the infected group (*P* < 0.001). Notably, the IL-1β expression for both infected groups were two-fold higher at 18 dpi compared with the rest of the timepoints and significantly higher (*P* < 0.001) than the level in the negative control group. There were no differences between infected groups.

## Discussion

The results of the present study revealed a significant increase in T regulatory cells (Foxp3^+^) detected by immunohistochemistry and gene expression in liver of both infected control (group 2) and immunized (group 1) sheep compared to the uninfected control group at 9 and 18 dpi. These results are in agreement with the significant increase of this cell type described in goats and sheep experimentally infected with *F. hepatica* at 9 dpi [12] and with the increase of T regulatory cells (Foxp3^+^) in the abomasum of sheep infected with *Teladorsagia circumincta* [11] and suggests that both parasites induce expansion of Foxp3^+^ T cells to modulate the host response facilitating their survival in host tissues. The dynamic of Foxp3 expression detected by immunohistochemistry (IHC) and by PCR was similar in the immunized and infected control groups, which was expected since the vaccine was non-protective. In *T. circumcincta* infected sheep, expansion of Foxp3^+^ T cells was associated with increased IL-10 gene expression [11]. The results of the present study revealed a very similar dynamic of IL-10 gene expression and Foxp3 expression of both antigens and gene as well as strong correlation between IL-10 and Foxp3 in the two infected groups, which is in agreement with previous results reported in sheep infected with *T. circumcincta* [11]. An increase in IL-10 has also been reported in peripheral blood mononuclear cell (PBMC) from sheep experimentally infected with *F. hepatica* during chronic infection stages, and levels were significantly higher in sheep with high fluke burdens [25]. Cattle naturally infected with *F. hepatica* also showed increased gene expression for IL-10 in liver tissues compared to uninfected control cattle [26], suggesting a role of this cytokine in modulating the host response facilitating *F. hepatica* survival during chronic infection. The results of the present study revealed that the role of increases in IL-10 modulating the host response is relevant since the early stages of the hepatic migratory stage.

Hepatic fibrosis is a typical lesion of chronic fasciolosis in sheep [27, 28] and TGF-β inducing SMAD signaling plays an important role in fibrosis progression [29]. The evaluation of portal fibrosis in Masson’s trichrome stained sections in the present study revealed that fibrosis is increased significantly both in the immunized and in the infected control groups at 9 and, particularly, at 18 dpi compared to the uninfected control. The lower fibrosis found at 9 and 18 dpi in group 1 respect to group 2 is in accordance with the higher gross hepatic lesion found at 9 dpi in the group 2 compared to the group 1 [13]. Portal fibrosis is a chronic lesion that take some days to develop, thus since at 9 dpi hepatic lesions from group 2 were more severe than in group 1, this may justify that at 9 and 18 dpi hepatic fibrosis was lower in group 1 than in group 2. The portal fibrosis intensify coincides with the increase of TGF-β gene expression in both infected groups, which occurs from 1 dpi onwards, and it was particularly high at 18 dpi. These results agree with the reported upregulation of the COL1A1 gene induced by TGF-β in PBMCs from *F. hepatica* infected sheep at 7 dpi [30] and at chronic stages of infection [25, 31]. Researchers have identified increases in the expression of several genes (SMAD3, SMAD4, COL1A1) related to TGF-β and associated with evidence of fibrosis-associated cell types, including fibroblast and stellate cell lines, in acute stages of infection in sheep.

Two of the most participative cytokines in the inflammatory reactions of the innate immune response in mammals are IL-1β and TNF-α, and are present in the acute stages of disease [32, 33]. IL-1β is responsible of the promotion of phagocyte activity favoring the host immune response against infection [34]. On the other hand, TNF-α is an important mediator of proinflammatory mechanisms [35]. A study showed that mice intraperitoneally injected with fatty acid binding protein (Fh12) from *F. hepatica* and sacrificed at 12 h post-infection, did not express proinflammatory cytokines. And mice injected with Fh12 and subsequently with bacterial lipopolysaccharide (LPS) resulted in significantly reduced levels of TNF-α, likewise in the same experiment they found suppression of the expression of IL-1β and TNF-α in cultivated cells from bone marrow previously treated with LPS and Fh12 [36]. Similar results were found in our study due to the fact that, at 1 dpi, low expression of mRNA for both cytokines were found in comparison with higher levels found at 3, 9, and 18 dpi. It was remarkable that at 1, 3, and 9 dpi gene expression for TNF-α was higher in group 1 (immunized) than in group 2 (infected control), despite the vaccine being non-protective. This finding agrees with the higher TNF-α expression in liver tissues from rats immunized with the myosin regulatory light chain (MRLC) of *F. hepatica* and partially protected compared to unimmunized rats in chronic infections, coinciding with lower hepatic lesions in immunized rats [37]. By contrast, in the present study, immunized sheep did not show fluke burden reduction, but a reduction of gross hepatic lesions was found at 9 dpi in immunized sheep compared to unimmunized and infected animals [13].

In summary, the results of the present study revealed no consistent differences in proinflammatory and regulatory cytokine expression in liver tissue from immunized (not protected) and unimmunized sheep infected with *F. hepatica*. The upregulation of Foxp3 jointly with the overexpression of IL-10 and TGF-β in both immunized and non- immunized sheep suggests that *F. hepatica* induces a modulation of the host response in the very early stages of infection to facilitate the parasite survival at these critical stages of the disease. Finally, the smaller fibrotic areas and lower levels of Foxp3^+^ cells (IHC and RT-PCR) found in the livers of the immunized and infected group as well the lower gross lesions found particularly at 9 dpi [13] suggest the vaccine induced partial but not sufficient protection. Deeper studies are needed to know the immune mechanisms involved at initial stages of this disease with the aim of developing an effective vaccine.

## Abbreviations

Foxp3: forkhead box P3 transcription factor
IL10: interleukin 10
TGFβ: transforming growth factor beta
TNFα: tumor necrosis factor alpha
IL1β: interleukin 1
qRT-PCR: real time polymerase chain reaction
rCL1: recombinant cathepsin L1
iNOS: inducible nitric oxide synthase
FhCL1: *Fasciola hepatica* cathepsin L1
ELISA: enzyme-linked immunosorbent assay
PBS: phosphate buffered saline
DEPC: diethylpyrocarbonate
bp: base pair
RIN: RNA integrity number
IHC: immunohistochemistry
PBMC: peripheral blood mononuclear cell
